# Assessing the suitability of video-assisted anal fistula treatment for obese patients compared to conventional surgery: a question worth investigating

**DOI:** 10.1007/s00384-024-04683-y

**Published:** 2024-07-15

**Authors:** Xiao-Li Tang, Zi-Yang Xu, Jun Yang, Zhe Yang, Zhi-Gang Wang, Zheng-Yun Zhang, Jing Yao

**Affiliations:** Department of Surgery, Shanghai Sixth Peoples Hospital Affiliated to Shanghai Jiao Tong University School of Medicine, Shanghai, 200233 China

**Keywords:** Anal fistula, Video-assisted anal fistula treatment, Cutting seton, Surgical outcomes, Recurrence

## Abstract

**Background and aims:**

Video-assisted anal fistula treatment (VAAFT) is an innovative surgical approach enabling the direct visualization of the fistula tract structure. This study aims to assess the efficacy of VAAFT in comparison with that of traditional surgical methods and explore potential risk factors contributing to fistula recurrence to provide new recommendations for surgical selection.

**Materials and methods:**

Information was collected from 100 patients with complex anal fistula (CAF) in our hospital who underwent surgical treatment from January 2021 to January 2023. We compared the baseline information and surgical outcomes of two groups, analyzed the risk factors for fistula recurrence by using logistic regression analysis, and conducted further exploration by using the body mass index.

**Results:**

Equal numbers of patients underwent VAAFT and traditional surgeries, and no significant differences in baseline information were observed. Patients who received VAAFT experienced less intraoperative bleeding (15.5 (14.0–20.0) vs. 32.0 (25.0–36.0)), shorter hospital stays (2.0 (2.0–2.5) vs. 3.0 (3.0–3.5)), reduced postoperative pain and wound discharge, but longer operative times (43.3 ± 6.9 vs. 35.0 (31.5–40.0)) compared with patients who underwent traditional surgeries. No significant differences in recurrence rates were found three and six months after operation (the *p*-values were 0.790 and 0.806, respectively). However, the Wexner scores of the VAAFT group were significantly low in the first follow-up (0 (0–1.0) vs. 2.0 (1.0–2.0)). Postoperative recurrence of fistulas may be associated with obesity (*p*-value = 0.040), especially in patients undergoing traditional surgeries (*p*-value = 0.036).

**Conclusion:**

VAAFT offers advantages, such as less pain, less trauma, and faster recovery, compared with traditional surgical treatment. Obese patients with CAF are prone to recurrence, and we recommend that they undergo VAAFT treatment rather than traditional surgeries.

**Supplementary Information:**

The online version contains supplementary material available at 10.1007/s00384-024-04683-y.

## Introduction

An anal fistula tract is an aberrant link between the anorectal canal and perianal skin and often accompanied with pain and discomfort. The external opening on the skin may continuously or intermittently discharge purulent secretions, and in severe cases, feces may even be discharged through the fistula tract [1]. The risk of the disease is higher in males than in females [2, 3]. When anal fistula presents recurrently with multiple tracts or involving a large portion of the sphincter, it is called complex anal fistula (CAF) [4].

The primary target of anal fistula treatment is to achieve healing, preserve the integrity of the anal sphincter, and minimize surgical trauma; accurately identifying the internal opening and thoroughly eradicating the fistula tract during the treatment process are crucial [5]. Surgery remains an effective method for treating anal fistula. However, traditional surgical approaches, such as fistulotomy and cutting seton, pose a high risk of recurrence and incontinence in CAF treatment [6, 7]. Advancements in minimally invasive surgical techniques and the emphasis on rapid recovery have motivated researchers to develop several novel treatment methods for reducing postoperative recurrence and fecal incontinence. These methods encompass procedures, such as ligation of the intersphincteric fistula tract (LIFT), adipose-derived stem cell augmentation, and fistula laser closure (FiLaC). These emerging technologies have shown considerable variations in cure rates across different studies [8]. Current treatment outcomes remain unsatisfactory.

Meinero et al. pioneered the integration of endoscopic techniques in the treatment of anal fistula and developed the video-assisted anal fistula treatment (VAAFT), which enables the direct visualization of the internal opening, thus facilitating precise localization [9]. Several studies have confirmed the efficacy of this technique, compared it with other treatment modalities, and obtained satisfactory results [10–12]. Torre et al. compared the outcomes of patients treated with VAAFT and LIFT and found that the recurrence rates of the two groups are similar [11]. In Liu et al.’s trial, the patients treated with VAAFT showed less pain and faster recovery compared with patients with CAF who received fistulotomy plus seton treatment [12]. However, further research is needed on this technique and the prognosis of anal fistula patients given that outcomes are influenced by various factors. Therefore, we conducted a preliminary comparison of VAAFT with traditional surgical treatment to identify new risk factors and provide useful references to anal fistula patients.

## Materials and methods

### Study population

Pertinent data from 100 consecutive cases of patients with CAF who underwent surgical treatment in our hospital from January 2021 to January 2023 were collected and analyzed in this retrospective study. All patients with CAF aged 18 and above were included. The patients were categorized into either the traditional surgical treatment group (conventional group) or the VAAFT group on the basis of the type of surgery they underwent. All patients did not present with acute onset symptoms and underwent a series of standardized clinical assessments, including ultrasound, digital rectal examination, and colonoscopy, to confirm the absence of contraindications to the surgical procedures used in this study and to assess the anal function. Patients with tuberculosis, inflammatory bowel disease, or suspected malignant tumors were excluded through preoperative colonoscopy examination. Additionally, individuals with recurrent transsphincteric anal fistula were excluded on the basis of medical records. Our study protocol was approved by the Ethics Committee of Shanghai Sixth People’s Hospital Affiliated to Shanghai Jiao Tong University School of Medicine, and all patients, who had the option to decline data retention for this study, provided informed consent. This study adhered to the reporting recommendations outlined in the STROBE guidelines from the Equator Network (https://www.equator-network.org/).

### Preoperative preparation, surgical procedures, and postoperative care

The patients underwent detailed medical history inquiries and clinical examinations to confirm the diagnosis of anal fistula. Imaging examinations were conducted to assess the fistula characteristics, including the number of tracts, locations, and internal openings. Bowel preparation was administered to all patients preoperatively, and the patients were given either spinal anesthesia or general anesthesia while assuming the lithotomy position during the procedure. The surgeries were conducted by an identical surgical team by using standardized techniques. The team members had received relevant training and had sufficient surgical experience.

In the conventional group, 24 patients with low anal fistula were managed with traditional fistulotomy (or fistulectomy). After anesthesia administration, a probe was inserted into the external opening, and the internal opening was located via a digital rectal examination. The probe was gently passed through both openings, after which the skin, subcutaneous tissue, internal fistula opening, and necrotic tissue were incised layer by layer. A curette was then used to meticulously remove the granulation tissue. Twenty-six patients with high anal fistula were treated with cutting seton (seton treatment). A probe was gently inserted through the external and internal openings of the fistula via a rectal examination. The portion of the fistula tract external to the sphincter was then opened along the probe, and granulation tissue was meticulously removed using a curette to achieve hemostasis. Subsequently, the seton was threaded through the fistula tract and tightened weekly until it passed through the sphincter.

The VAAFT group used equipment manufactured by Karl Storz SE-Tuttlingen (Germany). The equipment included a fistuloscope, forceps, a fistula brush, a closure device, and a monopolar electrode. The fistuloscope comprised an operating channel and an irrigation channel connected to a bag containing 1% mannitol–glycine solution. VAAFT was divided into two stages: diagnostic and operative. The diagnostic stage aimed to delineate the anatomical structure of the fistula tract, identify the internal opening, and detect any potential fistulae and abscess cavities. During this stage, the external opening was dilated appropriately, and the fistuloscope was gently advanced through it while the water pressure was adjusted to expand the fistula tract, thus ensuring optimal visualization within the lumen until the scope reached the end of the fistula tract. In most cases, the fistuloscope could be advanced from the internal opening to identify it, and two to three stitches were placed along its edge as markers. The operative stage aimed to completely obliterate the fistula tract and close the internal opening. The electrode was replaced and introduced through the surgical channel. Under direct visualization, continuous electrocoagulation was employed to destroy the fistula tract and any associated cavities and abscesses. Fistula brushes and forceps were utilized to excise fibrous necrotic tissue from the fistula tract, and continuous irrigation with irrigation fluid was performed to ensure the complete removal of detached necrotic tissue. Then, the internal opening was directly sutured closed, and thorough hemostasis was achieved.

During the perioperative period, antibiotics were not routinely administered to the patients in both groups, and postoperative analgesics were administered only when necessary. If a patient’s condition was stable and no complications were present, the patient could begin a liquid diet six hours after surgery. The patients were advised to take a warm sitz bath after defecation to cleanse the wound and alleviate pain.

### Exposure and outcome

Basic demographic information, including gender, age, body mass index (BMI), history of diabetes, and previous anal surgery, was collected from medical records. Patients with BMI > 28 were classified as obese, and the rest were classified as nonobese [13]. Fistula characteristics, such as the location of the fistula tract and the type, were confirmed through imaging examinations. The location of the fistula tract was classified as high and low depending on whether it passed through the upper portion of the deep external sphincter muscle or not [1]. The type of anal fistula was categorized using Park’s classification system, which is based on the anatomical location of the fistula tract and encompasses intersphincteric, transsphincteric, extrasphincteric, and suprasphincteric types [14]. Surgical-related information, including operative time and intraoperative blood loss, was collected.

Postoperative indicators encompassed length of hospital stay, pain intensity, postoperative complications, discharge status, Wexner score, and outcomes of healing and recurrence. Postoperative pain intensity was assessed using the Visual Analog Scale for Pain (VAS pain), which measures pain intensity on a scale of 0 to 10, with “no pain at all” and “pain as severe as possible” representing the two extremes [15]. Postoperative complications included postoperative bleeding and urinary retention, and the number of patients experiencing complications was recorded. Wound secretions were assessed on the second and fifth postoperative days and graded as 1, 2, and 3 on the basis of the extent of gauze infiltration, with uniform gauze dimensions [12]. Patients underwent two follow-up visits (three months and six months) postoperatively. The first follow-up visit was conducted in the outpatient clinic. Disappearance of symptoms and signs and wound healing without discharge were considered indicative of healing, whereas the reappearance of purulent discharge in previously healed wounds was considered recurrence. The Wexner score questionnaire was administered during the first follow-up visit to evaluate the presence and severity of fecal incontinence among patients [16]. The second assessment was conducted via telephone to investigate fistula recurrence six months after operation.

### Statistical analysis

Analysis was performed with SPSS version 27.0 (IBM, Armonk, NY, the USA). Gender, history of diabetes, previous anal surgery, anal fistula location, anal fistula type, postoperative complications, and postoperative discharge status were categorical variables reported as numbers and percentages. The remaining indicators were continuous variables and presented as mean ± standard deviation (SD). The differences in count data between the two groups were analyzed using the chi-square test or Fisher’s exact test, and the differences in measurement data were assessed using the independent sample Mann–Whitney U test or T-test. Logistic regression analysis was utilized to explore independent risk factors for postoperative fistula recurrence, and the results were reported as 95% confidence intervals (CIs) and odds ratios (ORs). A *p*-value < 0.05 was considered statistically significant for all analyses.

## Results

### Comparison of basic information

From January 2021 to January 2023, a total of 100 consecutive patients with CAF underwent surgical treatment, with 50 patients receiving traditional surgical treatment and 50 patients undergoing VAAFT. Table 1 presents the baseline information of the patients. No notable discrepancies were found between the two groups in terms of age (*p*-value = 0.499), gender (*p*-value = 0.640), BMI (*p*-value = 0.841), history of diabetes (*p*-value = 0.779), previous anal surgeries (*p*-value = 0.685), anal fistula location (*p*-value = 0.841), and Park’s classification (*p*-value = 1.000). In the VAAFT (78.0%) and conventional (74.0%) groups, the proportion of males exceeded that of females.

**Table 1:** Comparison of baseline characteristics between two groups

Variable		VAAFT (*N* = 50)	Conventional surgery (*N* = 50)	*p*-value
Age (year)				
	< 50	38 (76.0%)	35 (70.0%)	0.499
	≥ 50	12 (24.0%)	15 (30.0%)	
Sex				
	Male	39 (78.0%)	37 (74.0%)	0.640
	Female	11 (22.0%)	13 (26.0%)	
BMI				
	< 28	24 (48.0%)	25 (50.0%)	0.841
	≥ 28	26 (52.0%)	25 (50.0%)	
Diabetes				
	Yes	7 (14.0%)	8 (16.0%)	0.779
	No	43 (86.0%)	42 (84.0%)	
Previous anal surgery				
	Yes	22 (44.0%)	20 (40.0%)	0.685
	No	28 (56.0%)	30 (60.0%)	
Fistula location				
	High type	27 (54.0%)	26 (52.0%)	0.841
	Low type	23 (46.0%)	24 (48.0%)	
Park’s type				
	Inter-sphincteric	12 (24.0%)	11 (22.0%)	1.000
	Trans-sphincteric	25 (50.0%)	26 (52.0%)	
	Supra-sphincteric	9 (18.0%)	8 (16.0%)	
	Extra-sphincteric	4 (8.0%)	5 (10.0%)	

*VAAFT* video-assisted anal fistula treatment, *BMI* body mass index

### Comparison of postoperative efficacy between the two groups

The comparison of the postoperative results of the patients is shown in Table 2. No notable variance in postoperative complication rates, encompassing postoperative bleeding (*p*-value = 0.495) and postoperative urinary retention (*p*-value = 0.617), was observed between the two groups. Compared with the conventional group, the VAAFT group had lower intraoperative bleeding (15.5 (14.0–20.0) vs. 32.0 (25.0–36.0) mL, *p*-value < 0.001), shorter hospital stays (2.0 (2.0–2.5) vs. 3.0 (3.0–3.5) days, *p*-value < 0.001), but longer operating time (43.3 ± 6.9 vs. 35.0 (31.5–40.0) min, *p*-value < 0.001). We assessed the VAS pain scores of the patients on the first and third days after operation, and the pain intensity of the VAAFT group was low (*p*-value < 0.001 for both). Additionally, we evaluated wound secretion in the patients on the second and fifth days after operation, and the VAAFT group exhibited low secretion levels (*p*-value < 0.001 for both), with grade 1 being predominant (56.0%) in the VAAFT group and grade 2 (54.0%) being predominant in the conventional group on the second day. At the third month after operation, nine and eight cases of recurrence were observed in the VAAFT and conventional groups, respectively, with no significant difference in recurrence rate (*p*-value = 0.790). However, at this time, the Wexner score in the VAAFT group was significantly lower than that in the conventional group (0 (0–1.0) vs. 2.0 (1.0–2.0), *p*-value < 0.001). Six months after operation, the recurrence rates were 22.0% in the VAAFT group and 20.0% in the conventional group, with no significant difference (*p*-value = 0.806).

**Table 2:** Comparison of surgical outcomes between the two groups

Variable		VAAFT (*N* = 50)	Conventional surgery (*N* = 50)	*p*-value
Postoperative bleeding				
	Yes	0 (0)	2 (4.0%)	0.495
	No	50 (100.0%)	48 (96.0%)	
Urinary retention				
	Yes	1 (2.0%)	3 (6.0%)	0.617
	No	49 (98.0%)	47 (94.0%)	
Intraoperative blood loss (mL)				
	Median (IQR)	15.5 (14.0–20.0)	32.0 (25.0–36.0)	** < 0.001**
Postoperative hospital stays (days)				
	Median (IQR)	2.0 (2.0–2.5)	3.0 (3.0–3.5)	** < 0.001**
Operating time (min)				
	Mean ± SD/Median (IQR)	43.3 ± 6.9	35.0 (31.5–40.0)	** < 0.001**
Postoperative pain				
	1 day	3.0 (2.0–3.0)	4.0 (4.0–5.0)	** < 0.001**
	3 days	1.0 (1.0–1.3)	2.0 (2.0–3.0)	** < 0.001**
Wound secretion (2 days)				
	1	28 (56.0%)	11 (22.0%)	** < 0.001**
	2	18 (36.0%)	27 (54.0%)	
	3	4 (8.0%)	12 (24.0%)	
Wound secretion (5 days)				
	1	44 (88.0%)	27 (54.0%)	** < 0.001**
	2	6 (12.0%)	17 (34.0%)	
	3	0 (0)	6 (12.0%)	
Wexner score (3 months)				
	Median (IQR)	0 (0–1.0)	2.0 (1.0–2.0)	** < 0.001**
Curative effect (3 months)				
	Healing	41 (82.0%)	42 (84.0%)	0.790
	Recurrence	9 (18.0%)	8 (16.0%)	
Curative effect (6 months)				
	Healing	39 (78.0%)	40 (80.0%)	0.806
	Recurrence	11 (22.0%)	10 (20.0%)	

*VAAFT* video-assisted anal fistula treatment, *IQR* interquartile range, *SD* standard deviation

### Exploring the risk factors for anal fistula postoperative recurrence

Univariate logistic regression analysis was conducted with postoperative recurrence at six months as the outcome variable, and the results (Table 3) indicated that obesity (OR = 2.986, 95% CI: 1.050–8.492, *p*-value = 0.040) might be correlated with postoperative recurrence of anal fistula. However, other factors, including treatment (*p*-value = 0.950), age (*p*-value = 0.464), gender (*p*-value = 0.551), history of diabetes (*p*-value = 0.211), anal fistula location (*p*-value = 0.949), and operating time (*p*-value = 0.484), were not significantly associated with postoperative recurrence. To exclude the influence of confounding factors, we conducted multivariate regression analysis and found that obesity (OR = 2.989, 95% CI: 1.011–8.837, *p*-value = 0.048) was still a risk factor for the prognosis of patients under the influence of confounding factors. The patients were stratified based on BMI into two: BMI ≥ 28 and BMI < 28 groups. In the overall cohort, the recurrence rate was 29.4% in the BMI ≥ 28 group and 12.2% in the BMI < 28 group, indicating a significant difference in recurrence rates six months after operation (*p*-value = 0.036). Further subgroup analysis of the VAAFT and conventional groups revealed no significant difference in recurrence rates between obese and nonobese patients undergoing VAAFT (*p*-value = 0.387), whereas obese patients undergoing traditional surgical treatment exhibited a high recurrence rate six months after operation (*p*-value = 0.036) (Table 4). Therefore, on the basis of the recurrence on the sixth month after surgery, we further performed logistic regression analysis on the patients in the conventional group, and the analysis results (Table 5) indicated that obesity (OR = 5.412, 95% CI: 1.017–28.791, *p*-value = 0.048) may be associated with recurrence in this subgroup; the same conclusion was suggested after the confounders were excluded (OR = 6.356, 95% CI: 1.104–36.584, *p*-value = 0.038). In-depth analysis of the patients with BMI ≥ 28 revealed that among the obese individuals, the different surgical treatments did not significantly affect postoperative recurrence at 3 and 6 months (with *p*-values of 0.682 and 0.694, respectively). However, the patients who underwent VAAFT surgery experienced reduced postoperative pain and good preservation of the anal sphincter function (*p*-values < 0.001, Table 6).

**Table 3:** Logistic analysis of risk factors for fistula recurrence after surgery

Variable	Univariate		Multivariate	
	OR (95% CI)	*p*-value	OR (95% CI)	*p*-value
Treatment (Conventional vs. VAAFT)				
	1.128 (0.431–2.956)	0.806	1.036 (0.342–3.141)	0.950
Age (≥ 50 vs. < 50)				
	1.475 (0.522–4.171)	0.464	1.475 (0.522–4.171)	0.930
Sex (Male vs. Female)				
	1.441 (0.433–4.790)	0.551	1.466 (0.424–5.068)	0.546
Diabetes (Yes vs. No)				
	2.156 (0.647–7.183)	0.211	2.178 (0.519–9.149)	0.288
BMI (≥ 28 vs. < 28)				
	2.986 (1.050–8.492)	**0.040**	2.989 (1.011–8.837)	**0.048**
Fistula location (High vs. Low)				
	0.969 (0.370–2.540)	0.949	1.020 (0.365–2.851)	0.969
Operating time (≥ 40 vs. < 40)				
	1.435 (0.522–3.945)	0.484	1.163 (0.361–3.745)	0.800

*VAAFT* video-assisted anal fistula treatment, *BMI* body mass index

**Table 4:** Comparison of postoperative outcomes stratified by BMI groups

Variable		Overall cohort (*N* = 100)			VAAFT (*N* = 50)			Conventional surgery (*N* = 50)		
		BMI ≥ 28 (*N* = 51)	BMI < 28 (*N* = 49)	*p*-value	BMI ≥ 28 (*N* = 26)	BMI < 28 (*N* = 24)	*p*-value	BMI ≥ 28 (*N* = 25)	BMI < 28 (*N* = 25)	*p*-value
Postoperative pain										
	1 day	4.0 (3.0–4.0)	4.0 (3.0–5.0)	0.441	3.0 (2.0–3.0)	3.0 (2.0–3.0)	0.874	4.0 (4.0–5.0)	5.0 (4.0–5.0)	0.281
	3 days	2.0 (1.0–2.0)	2.0 (1.0–3.0)	0.650	1.0 (1.0–1.3)	1.0 (1.0–1.8)	0.754	2.0 (2.0–3.0)	3.0 (2.0–3.0)	0.787
Wexner score										
	3 months	1.0 (0–2.0)	1.0 (0–2.0)	0.426	0.5 (0–1.0)	0 (0–1.0)	0.516	2.0 (1.0–2.0)	2.0 (1.0–2.0)	0.302
Curative effect (3 months)										
	Healing	40 (78.4%)	43 (87.8%)	0.217	21 (80.8%)	20 (83.3%)	0.815	19 (76.0%)	23 (92.0%)	0.127
	Recurrence	11 (21.6%)	6 (12.2%)		5 (19.2%)	4 (16.7%)		6 (24.0%)	2 (8.0%)	
Curative effect (6 months)										
	Healing	36 (70.1%)	43 (87.8%)	**0.036**	19 (73.1%)	20 (83.3%)	0.387	17 (68.0%)	23 (92.0%)	**0.036**
	Recurrence	15 (29.4%)	6 (12.2%)		7 (26.9%)	4 (16.7%)		8 (32.0%)	2 (8.0%)	

*BMI* body mass index, *VAAFT* video-assisted anal fistula treatment

**Table 5:** Logistic analysis of risk factors for fistula recurrence after conventional treatment

Variable	Univariate		Multivariate	
	OR (95% CI)	*p*-value	OR (95% CI)	*p*-value
Age (≥ 50 vs. < 50)				
	1.000 (0.220–4.536)	1.000	0.628 (0.073–5.403)	0.672
Sex (Male vs. Female)				
	1.517 (0.278–8.287)	0.630	1.945 (0.306–12.379)	0.481
Diabetes (Yes vs. No)				
	1.417 (0.240–8.367)	0.701	1.682 (0.127–22.255)	0.693
BMI (≥ 28 vs. < 28)				
	5.412 (1.017–28.791)	**0.048**	6.356 (1.104–36.584)	**0.038**
Fistula location (High vs. Low)				
	0.905 (0.226–3.619)	0.887	1.147 (0.236–5.566)	0.865
Operating time (≥ 40 vs. < 40)				
	0.902 (0.220–3.702)	0.886	0.896 (0.197–4.081)	0.888

*BMI* body mass index

**Table 6:** Comparison of postoperative indicators between two groups of patients with BMI ≥ 28

Variable		VAAFT (*N* = 26)	Conventional surgery (*N* = 25)	*p*-value
Postoperative pain				
	1 day	3.0 (2.0–3.0)	4.0 (4.0–5.0)	** < 0.001**
	3 days	1.0 (1.0–1.3)	3.0 (2.0–3.0)	** < 0.001**
Wexner score				
	3 months	0.5 (0–1.0)	2.0 (1.0–2.0)	** < 0.001**
Curative effect (3 months)				
	Healing	21 (80.8%)	19 (76.0%)	0.682
	Recurrence	5 (19.2%)	6 (24.0%)	
Curative effect (6 months)				
	Healing	19 (73.1%)	17 (68.0%)	0.694
	Recurrence	7 (26.9%)	8 (32.0%)	

*VAAFT* video-assisted anal fistula treatment

## Discussion

Low anal fistulas treated with fistulotomy typically exhibit a high cure rate, but surgical treatment of high anal fistulas remains a major challenge because of the risk of incontinence when more than one-third of the anal sphincter is involved [17]. Cutting seton treatment is a widely used and effective method for surgically managing high anal fistulas. The seton aids in pus drainage, stimulates fibrosis, gradually cuts off the fistula, and consequently promotes healing [18]. However, a summary analysis of studies suggested that the average incontinence rate after cutting seton treatment is approximately 12.0% [19]. Moreover, a study reported that more than half of its 35 reviewed patients experienced mild anal injury symptoms after receiving cutting seton treatment [20]. In the past few years, the emergence of several new technologies has provided new insights into the treatment of anal fistulas. LIFT involves ligation and division of the fistula and internal opening at the level of the anal sphincter and removal of potentially infected glands [21]. FiLaC uses a laser probe to destroy and clear the fistula tract [22]. The average success rate of LIFT is approximately 76.5%, and that of FiLaC is 71%–82% [8]. Both techniques use external injection of methylene blue or hydrogen peroxide to identify internal openings, similar to traditional fistulotomy and cutting seton treatments. However, because internal openings cannot be directly observed, the risk of overlooking fistula branches and narrow internal openings exists.

VAAFT overcomes the limitations of the aforementioned techniques by irrigating fistula secretions and providing visualization through a fistuloscope, thus enabling an improved detection of potential fistula branches and internal openings. Studies have demonstrated the superiority of VAAFT in identifying internal openings [23, 24]. Accurate identification of fistula branches and internal openings may help reduce the recurrence rate of anal fistulas. Our follow-up data indicated that the recurrence rates of 50 patients who underwent VAAFT three and six months after operation were 18% and 22%, respectively, which are similar to the recurrence rates in previous studies [25] and comparable to the recurrence rates of traditional surgical treatment in our study. However, the postoperative incontinence rate after VAAFT was low, suggesting relatively minor surgical trauma and good protection of the sphincter function, which enhance patients’ future quality of life. Furthermore, our research indicated that VAAFT offers advantages in reducing intraoperative bleeding, shortening hospital stay, relieving postoperative pain, and expediting wound recovery.

The absence of a significant difference in cure rates between VAAFT and traditional surgical treatment may be attributed to the following reasons. Given the anatomical structure of the pelvis, fistula tracts are often curved and can manifest in various shapes and sizes [14]. Consequently, when the fistula tract is too narrow, contains multiple curves, or exhibits a large curvature angle, rigid fistuloscopes may not be able to thoroughly explore the entire fistula tract, posing challenges in accurately identifying internal openings, a phenomenon that is consistent with our surgical experience. Adequate closure of internal openings and effective drainage are also crucial for the success of VAAFT. Currently, the methods used to close internal openings include suturing, staplers, advancement flap techniques, over-the-scope clips (OTSCs), fibrin glue injection, and fistula plugs, but evidence is insufficient to determine which method of internal closure has a distinct advantage over the others. Some researchers suggested that in most cases, OTSC is preferable to staplers, and in challenging scenarios, advancement flap techniques may be the only option despite the elevated risk of reinfection in such instances [26]. The long-term success rate of fibrin glue injection is disappointing, prompting some studies to propose the utilization of new adjunctive agents, such as platelet-rich fibrin glue, which has produced promising results [27]. However, further research is required to validate its reliability.

We conducted a risk analysis and found a significant correlation between obesity and postoperative recurrence of CAF. Further stratified analysis of the VAAFT and conventional groups revealed a high recurrence rate among the obese individuals in the conventional group, but obesity did not significantly affect recurrence in the patients undergoing VAAFT. This finding suggests new recommendations regarding the selection of surgical approaches for anal fistula patients with different BMIs, with obese individuals exercising great caution when they consider traditional surgical treatment. We believe that compared with traditional surgical methods, VAAFT treatment may be a preferable option for obese patients with CAF. Consequently, we conducted a thorough analysis of patients with BMI ≥ 28. We found no significant difference in recurrence rates between the two surgical approaches. However, the patients who underwent VAAFT treatment experienced reduced pain and exhibited minimal damage to the anal sphincter function, providing some support for our recommendation. Other possible risk factors demonstrated in literature, including lengthened operating times [12], male gender [28], and old age [29], were not validated in this study. Further analysis is needed to elucidate the reasons behind this phenomenon, potentially facilitate the selection of personalized treatment modalities for patients, and improve prognosis. Adipose tissue in obese individuals releases abundant proinflammatory signals, which may result in tissue damage [30]. Local perfusion of adipose tissue may be insufficient, thereby affecting wound healing. These factors may contribute to the high recurrence rate in obese patients after operation, with VAAFT causing less damage and being less influenced by these factors compared with traditional surgical treatment. However, these claims are currently speculative, and the specific mechanisms remain to be studied.

This study has several limitations. Our data were obtained through retrospective analysis at a single center, possibly introducing selection bias. As a newly introduced technique, VAAFT may be associated with lower surgical proficiency and less experienced doctors compared with traditional surgical treatment, and this could contribute to the relatively long duration of VAAFT procedures. We did not examine additional potential risk factors and confounding variables that were not included in this study. The comparison of the recurrence rates in the second and third follow-up visits showed that the recurrence rate tends to stabilize three months after surgery, albeit with some fluctuations. Long follow-up periods may be necessary to verify the recurrence rates of these surgical methods. Ultimately, we could not establish, on the basis of postoperative recurrence rates alone, that VAAFT is a superior surgical option for obese patients. Such establishment may necessitate a large sample size, extended follow-up duration, or multicenter experimental validation.

## Conclusion

Our study found that compared with traditional surgical treatment, VAAFT produces superior outcomes, including reduced pain, minimal damage, accelerated recovery, and improved preservation of the anal sphincter function in patients with anal fistula. Postoperative recurrence, particularly after traditional surgical treatment, may be associated with obesity. We suggest that compared with traditional surgery, VAAFT treatment may be more suitable for obese patients with CAF. Future technological advancements are expected to enhance the outcomes of VAAFT, thus enabling the selection of personalized and evidence-based treatment plans tailored to individual patients.

## Supplementary Information

Below is the link to the electronic supplementary material.

Supplementary file1 (PDF 18 KB)

## Data Availability

No datasets were generated or analysed during the current study.
